# Prognostic Association between Injury Severity Score and the Outcomes of Elderly Patients with Trauma in South Korea

**DOI:** 10.3390/jpm14070674

**Published:** 2024-06-23

**Authors:** Jae-Guk Kim, Hyun-Young Choi, Gu-Hyun Kang, Yong-Soo Jang, Wonhee Kim, Yoonje Lee, Chiwon Ahn

**Affiliations:** 1 Department of Emergency Medicine, Kangnam Sacred Heart Hospital, Hallym University College of Medicine, Seoul 07441, Republic of Korea; gallion01@hallym.ac.kr (J.-G.K.); emkang@hallym.or.kr (G.-H.K.); amicoys@hallym.or.kr (Y.-S.J.); wonsee02@hallym.or.kr (W.K.); yoonje@hallym.or.kr (Y.L.); 2 Department of Emergency Medicine, College of Medicine, Chung-Ang University, Seoul 06974, Republic of Korea; cahn@cau.ac.kr

**Keywords:** geriatrics, trauma, injury severity score, prognosis

## Abstract

This study investigated the impact of the Injury Severity Score (ISS) on treatment approaches and survival outcomes in trauma patients, focusing on comparing elderly (≥65 years) with non-elderly patients. It analyzed adult trauma cases with abnormal Revised Trauma Scores from January to December 2019, categorizing patients into three severity groups based on ISS: mild (1–8), moderate (9–15), and severe (≥16). The study examined how ISS influenced therapeutic interventions and survival among elderly patients, comparing these outcomes to non-elderly patients using multivariable logistic regression analysis. In 16,336 adult trauma cases out of 52,262 patients, including 4886 elderly and 11,450 non-elderly patients, findings revealed that in the severe group, elderly patients had a lower, though not statistically significant, incidence of surgical or embolization interventions compared to the moderate group, differing from non-elderly patients. No significant differences were observed in the mild group between elderly and non-elderly patients. However, elderly patients had higher intervention rates in the moderate group and lower in the severe group, with significantly lower survival-to-discharge rates in the severe group. The ISS is insufficient for assessing trauma severity in elderly patients. Additional tools are needed for better evaluation and treatment decisions.

## 1. Introduction

Trauma remains a significant public health concern worldwide, affecting individuals across all age groups [1]. However, with the global population experiencing a notable increase in elderly individuals, healthcare systems face unique challenges in terms of providing effective and tailored care to this growing demographic [2,3]. Understanding the prognostic factors specific to elderly patients with trauma is crucial for improving their outcomes and optimizing resource allocation in healthcare settings [2,4]. Similar to the demographic reality in many other countries, the population in South Korea is aging rapidly [2]. The unique characteristics of the elderly population in this country, including physiological changes, comorbidities, and functional limitations, justify a focused investigation into the prognostic factors that can influence their clinical outcomes [2,5,6].

One crucial aspect of prognostication in trauma care is the evaluation of injury severity. Injury Severity Scores (ISSs) are widely used to quantify the severity of traumatic injuries and provide standardized measures for risk stratification and prognostication [7]. A high ISS is generally associated with more severe injuries, higher mortality rates, and worse clinical outcomes. ISSs play a crucial role in the initial assessment and management of patients with trauma, as well as medical research on the subject and decision-making regarding the allocation of medical resources [8,9]. In geriatric patients with trauma, the etiologies of injury, physiological reactions, and both immediate and extended prognostic outcomes diverge significantly from those observed in non-elderly patients. Consequently, this population tends toward either in-hospital mortality or discharge with an optimistic prognostication of functional recovery [5,6]. However, it remains unclear how ISS and other prognostic factors differ in their predictive value when applied specifically to elderly patients with trauma vs. their non-elderly counterparts [7].

Despite therapeutic options for trauma patients typically being determined based on both anatomical and physiological abnormalities, this study aims to evaluate the value of the ISS as an adjunctive tool for medical decision-making and outcome prediction for elderly trauma patients, with a focus on comparing these patients to their non-elderly counterparts.

## 2. Materials and Methods

### 2.1. Study Design and Settings

This study was a retrospective analysis based on data from the 2018 Community-Based Severe Trauma Survey conducted in South Korea, which was compiled and subsequently disseminated by the Korea Disease Control and Prevention Agency (KDCA) following approval by Statistics in 2019. The dataset includes information regarding patients who sustained trauma injuries and were subsequently transported to hospital by the Korean Emergency Medical Services (EMS) department between January and December of 2018.

The Korean EMS operates under the purview of the National Emergency Management Agency, a governmental entity tasked with providing advanced cardiovascular and basic life support across its 17 provincial jurisdictions. Their fleet of ambulances is uniformly outfitted with automated external defibrillators and staffed by 2–3 emergency medical technicians who are trained in the administration of intravenous fluids and capable of performing advanced airway management techniques such as endotracheal intubation.

This study was approved by the institutional review board (IRB) of Kangnam Sacred Heart Hospital in 2022 (IRB approval no.: 2022-06-004). Owing to the retrospective design of the study and the use of deidentified clinical data, the requirement for informed patient consent was waived. The study’s methodological approach aligned with the guidelines stipulated in the Strengthening the Reporting of Observational Studies in Epidemiology checklist for observational studies.

### 2.2. Data Source

A community-based survey of severe trauma was conducted using medical records from hospitals previously affiliated with the Korea National Fire Agency. The records were anonymized by the Korean Personal Information Protection and Statistics Act. To ensure the reliability of these medical records, a community-based investigator conducted on-site verifications at each of the medical institutions. Data validation was performed by the KDCA. The survey encompassed a variety of variables, including demographic details, injury specifics, medical interventions, and patient outcomes post-hospitalization.

To assess the severity of trauma experienced by individuals, two established metrics were used: the Revised Trauma Score (RTS) and the ISS. The RTS, particularly favored for its utility in pre-hospital triage, calculates severity based on three critical parameters: the Glasgow Coma Scale score; systolic blood pressure; and respiratory rate. Its scores range from 0 to 12. A maximum score of 12 indicates the presence of normal vital parameters [10]. In the pre-hospital phase, emergency medical technicians (EMTs) are tasked with evaluating and documenting all patient information that is relevant to calculating these parameters. An RTS score of <12 is automatically assigned if a patient has a systolic blood pressure <90 mmHg, a respiratory rate outside the range of 10 to 29 breaths/min or altered levels of consciousness. This scoring mechanism aids EMS providers in determining the severity of a patient’s trauma. Conversely, the ISS is mostly used to evaluate progress in patients with trauma during the hospital phase [11]. ISS calculations make use of the Abbreviated Injury Scale, which categorizes the body into six sections (head, face, chest, abdomen, limbs, and external), with each assigned a severity score ranging from 0 (no injury) to 5 (severe injury). The ISS is derived by summing the highest scores from three of these six bodily sections, thus providing a comprehensive measure of injury severity [12]. Patients with trauma who initially presented with abnormal RTS values, as assessed by the attending EMTs, were considered for inclusion in our study.

### 2.3. Study Population

Adult patients with trauma (aged ≥ 18 years) who presented with abnormal RTSs between January and December of 2018 were enrolled. Those aged ≥ 65 years were classified as elderly [2,13]. Based on ISSs, the patients were stratified into three categories: mild (1–8), moderate (9–15), and severe (≥16) [14]. Specific criteria were used to exclude certain subsets of patients with trauma from the study population. Patients were also excluded if they had unknown or normal RTS values, were <18 years of age, had unknown ISSs, or had missing data regarding surgical interventions, embolization, or survival.

### 2.4. Variables

Patient data regarding injuries and outcome parameters were collected from the trauma registry. Various covariates were recorded—including patient sex, age, location, duration from emergency call until transfer to hospital, hospital type (i.e., level), mechanism of injury (e.g., transportation incidents, falls and sliding, contusion, penetration, or machine injury), ISS, and hospital management approach (e.g., transfusion, surgical treatment, or embolization). A detailed classification of the causes of trauma is provided in Table S1. Hospital levels were defined as first, second, third, and trauma centers (Table S2) [15,16]. The severity of trauma was classified into three groups based on the ISS range: mild (1–8); moderate (9–15); and severe (≥16) [14].

### 2.5. Outcome Measures

The primary outcome was the rate of surgery or embolization. The secondary outcome was survival until hospital discharge. If patients were transferred to another hospital, their outcome data were collected from the medical records of the second hospital.

### 2.6. Statistical Analysis

Descriptive statistics were used to summarize continuous data, with demographic features presented as medians and interquartile ranges. Categorical data were summarized as frequencies and percentages. The normality of each continuous variable was assessed using the Kolmogorov–Smirnov test. Pearson’s Chi-squared or Fisher’s exact tests were used to compare categorical variables, while the independent *t*-test for the parametric data and the Mann–Whitney U test for the nonparametric data were used to compare continuous ones.

For our multivariable regression analysis, any variables identified as having significance levels of *p* < 0.05 in the univariable analyses were included as covariates. Adjusted odds ratios (ORs) and their corresponding 95% confidence intervals (CIs) were calculated using multivariable logistic regression. A stepwise backward-elimination approach was used in our multivariable logistic regression model. All statistical analyses were conducted using SPSS version 26.0 software (IBM Corp., Armonk, NY, USA) and R package version 3.3.2. Statistical significance was defined as a *p*-value of <0.05.

## 3. Results

### 3.1. Patient Characteristics

**Figure 1:**
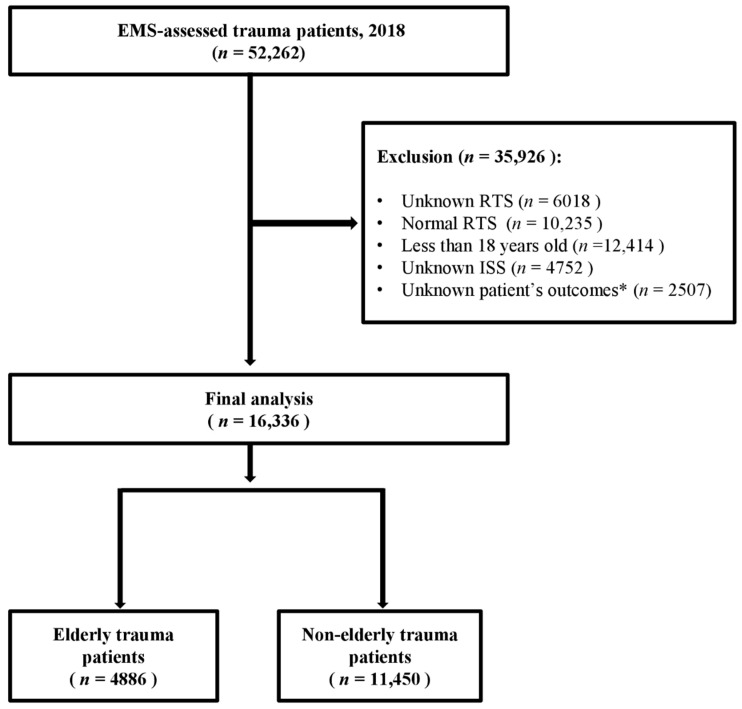
Flow chart of patient selection for this study. EMS, emergency medical system; RTS, revised trauma score; ISS, injury severity score. * Excluded because of unavailable medical records or transfer to another hospital.

Among the 52,262 EMS-assessed patients with trauma, 35,926 were excluded because of unknown RTS values (n = 6018), normal RTS values (n = 10,235), they were <18 years of age (n = 12,414), had unknown ISS values (n = 4752) or lacked outcome data (n = 2507). This left a total of 16,336 adult patients for our final analysis. These patients were further divided into two categories: elderly (aged ≥ 65 years; n = 4886) and non-elderly (aged < 65 years; n = 11,450; Figure 1).

The baseline characteristics of the patients with trauma are presented in Table 1. The proportion of male patients was significantly lower in the elderly group (57.2%) vs. the non-elderly one (72.1%; *p* < 0.001). A significantly higher percentage of elderly patients with trauma were from metropolitan areas (48.3%) compared to the non-elderly ones (42.5%; *p* < 0.001). Hospital-level care distribution varied slightly, with elderly patients being slightly less likely to be treated in Level III or trauma centers than their non-elderly counterparts (*p* = 0.026). The mechanisms of injury also differed significantly between the groups. The elderly patients were more likely to be injured by falls (56.3% vs. 38.3% in the non-elderly group, *p* < 0.001), whereas the non-elderly patients had higher incidence rates of traffic accidents (44.7% vs. 36.7% in the elderly group; *p* < 0.001) and penetrating or mechanical injuries (8.6% vs. 3.4% in the elderly group). In terms of injury severity, the elderly patients were found to have a higher percentage of severe injuries (ISS ≥ 16), at 16.6%, compared to 14.0% in the non-elderly group (*p* < 0.001). Transfusion rates were also higher in elderly patients (12.6% vs. 9.8% in the non-elderly group; *p* < 0.001). Both groups underwent similar rates of surgery and embolization procedures. Survival to discharge was significantly lower in the elderly group (84.3% vs. 93.5% in the non-elderly patients; *p* < 0.001).

### 3.2. Multivariable Logistic Analysis of the Rate of Surgery or Embolization and Survival at Hospital Discharge in Elderly Patients, according to Trauma Severity

Patients with ISSs of 9–15 were found to have 8.298× higher odds of requiring surgical or embolization interventions compared to those with ISSs of 1–8, which was statistically significant (95% CI: 6.856–10.043, *p* < 0.001). Notably, when comparing patients with ISSs of ≥ 16 to those with ISSs of 9–15, the odds ratio decreased to 0.847, although this was not statistically significant (95% CI: 0.690–1.040, *p* = 0.113; Table 2).

Regarding survival to discharge, patients with ISSs of 9–15 had significantly lower odds of surviving to discharge from the hospital compared to those with ISSs of 1–8 (OR = 0.143, 95% CI: 0.103–0.199, *p* < 0.001). This trend was even more pronounced for patients with ISSs of ≥16, where the odds of survival to discharge decreased even further, to 0.019 (95% CI: 0.013–0.026, *p* < 0.001) compared to the lowest ISS group. When the reference group was changed to those with ISSs of 9–15, the patients with ISSs of ≥16 still had significantly lower odds of survival (OR = 0.130, 95% CI: 0.102–0.165, *p* < 0.001; Table 2).

### 3.3. Multivariable Logistic Analysis of the Rate of Surgery or Embolization and Survival until Discharge in the Non-Elderly Patient Group, according to Trauma Severity

Regarding rate of surgery or embolization, patients with ISSs of 9–15 were significantly more likely to have an OR of 7.373 (95% CI: 6.443–8.436, *p* < 0.001), indicating a >7× increase in the odds of requiring these interventions compared to the reference group (ISS 1–8). For patients with ISSs of ≥ 16, the odds increased significantly—to an OR of 13.242 (95% CI: 11.291–15.532, *p* < 0.001)—suggesting an increase of >13× compared to the reference group (ISS 1–8). When the reference group was changed to those with ISSs of 9–15, patients with ISSs of ≥16 still showed a significantly higher likelihood of requiring surgical interventions or embolization, with an OR of 1.796 (95% CI: 1.530–2.109, *p* < 0.001; Table 3).

Regarding survival to discharge, our analysis also revealed a significant impact of ISS on survival to discharge. With an ISS of 1–8 as the reference, patients with ISSs of 9–15 had significantly reduced odds of survival, as indicated by an OR of 0.082 (95% CI: 0.055–0.120, *p* < 0.001). This trend was even more pronounced for those with ISSs of ≥16, who had an OR of 0.015 (95% CI: 0.010–0.022, *p* < 0.001), signifying a near-total decrease in the likelihood of survival compared to the reference group. When the reference group was changed to those with ISSs of 9–15, patients with ISSs of ≥16 had significantly lower odds of survival, with an OR of 0.183 (95% CI: 0.146–0.229, *p* < 0.001), highlighting the significant impact of higher injury severity on patient outcomes (Table 3).

### 3.4. Multivariable Logistic Analysis of the Rate of Surgery or Embolization and Survival to Discharge from the Hospital in Elderly Patients Compared to the Non-Elderly Ones, according to Trauma Severity

Regarding the rate of surgery or embolization, elderly patients with ISSs between 9 and 15 were found to have significantly increased odds (OR = 1.351, 95% CI: 1.035–1.763, *p* = 0.027) of requiring these interventions compared to non-elderly patients, indicating a higher vulnerability to moderate injuries in this demographic. Conversely, when the injury severity was ISS ≥ 16, elderly patients showed a decreased likelihood (OR = 0.623, 95% CI: 0.465–0.834, *p* = 0.002) of requiring surgery or embolization (Table 4).

Regarding survival to discharge, we found a consistent trend across all injury severity levels, with elderly patients generally showing lower odds of survival compared to non-elderly ones. Notably, for injuries with ISSs of ≥ 16, elderly patients had significantly lower odds of survival (OR = 0.705, 95% CI: 0.522–0.953, *p* = 0.023). However, for less severe injuries (ISS 1–8 and 9–15), the differences in survival odds were not statistically significant (Table 4).

## 4. Discussion

Our analysis showed that ISS correlates with both therapeutic approach and survival in elderly patients with trauma. When comparing elderly patients by varying levels of trauma severity, the incidence of surgery or embolization was lower in the severe group (ISS ≥ 16), although this was not statistically significant when this group was compared to the moderate group (ISS 9–15), indicating different outcomes from those observed in non-elderly patients with trauma. When evaluating the outcomes between elderly and non-elderly patients with trauma across the three severity groups, no discernible differences emerged within the mild group (ISS 1–8). Regarding surgical or embolization interventions, an increase was noted in the moderate group (ISS 9–15), whereas a significant decrease was observed in the severe group (ISS ≥ 16), indicating contrasting trends. Our analysis of survival to discharge revealed that in the group with severe trauma (ISS ≥ 16), elderly patients exhibited a lower likelihood of surviving until discharge from the hospital.

The mortality rate from trauma is higher, hospitalization periods are longer, and complications are more severe in elderly patients, even though the likelihood of sustaining serious injuries is lower than in non-elderly individuals [17]. The characteristics of aging, which vary based on socioeconomic and sociocultural contexts, typically involve decreased mental activity, impaired perception, reduced attention, diminished sensory functions such as vision and hearing, delayed reflexes, general muscle weakness, and movement disorders. Collectively, these factors increase the risk of trauma exposure among elderly adults [13,18]. In elderly patients with trauma, these changes result in different mechanisms of injury compared to non-elderly patients. Accidents and falls, which are common among elderly adults, often lead to more frequent fractures and higher rates of complications, hospitalization, and mortality. Trauma scoring systems such as the RTS and ISS are useful for triage, prognosis, predicting outcomes, and appropriately allocating resources for trauma care. However, the effectiveness of these scoring systems can vary when applied to elderly populations [19,20,21].

Vassallo et al. reported that the rate of life-saving interventions received by an adult trauma patient is strongly correlated with increasing ISS, but in this study, elderly patients showed different patterns [14]. Our analysis showed that, as ISS increased, the OR for surgery or embolization first increased, then decreased significantly, indicating a nuanced relationship between injury severity and the likelihood of receiving surgical treatment (Table 2). The information presented in Table 2 shows the impact of injury severity on outcomes in elderly patients with trauma when different ISS reference groups are compared. These data showed that changing the reference group significantly affected the OR for the likelihood of a patient undergoing surgery or embolization, as well as their survival rates until discharge. This underscores the crucial role of injury severity when determining patient outcomes, suggesting that, although more severe injuries increase the need for operative interventions or embolization, the benefits of these procedures may plateau or diminish in patients with the most severe injuries. It can thus be hypothesized that the frequency of surgical interventions among elderly patients declines as trauma severity increases. This trend likely results from the increased surgical risks associated with severe injuries. In addition, elderly patients with trauma commonly exhibit multiple comorbidities, further exacerbating the risk of adverse outcomes following surgery or other invasive procedures [22]. These comorbidities significantly affect clinical decision-making concerning the advisability of surgical or other invasive interventions. Table 3 illustrates similar trends among non-elderly patients, in which higher ISS correlated with a greater likelihood of undergoing surgical or embolization procedures. Survival rates also decreased significantly as ISS increased. The findings in Table 2 and Table 3 highlight the need for tailored approaches to trauma care in elderly patients that consider the unique vulnerabilities and treatment needs of this demographic, particularly at different levels of injury severity.

This study also highlighted the varying effects of aging on trauma outcomes across all three trauma severity groups analyzed (Table 4). Elderly patients with trauma exhibited an increased rate of requiring surgery or intervention compared to their non-elderly counterparts within the ISS 9–15 group. Conversely, in the group with severe trauma (ISS ≥ 16), elderly patients showed a decreased likelihood of receiving invasive interventions than non-elderly ones did. Regarding survival to discharge, elderly patients with trauma in the severe group (ISS ≥ 16) had significantly lower odds of survival compared to their non-elderly counterparts. Possible reasons for the varying impacts of old age on trauma outcomes include undefined trauma locations, which make it difficult to determine the severity of trauma. This issue arises because ISS only considers one injury in each body region. This leads to injuries being overlooked and to less severe injuries occurring in other body regions being included in the calculation over more serious ones in the same body region [23] and does not specifically identify whether surgeries are performed for head, thoracic, or abdominal injuries; thus, the severity of trauma can vary even between patients with the same ISS score [24]. Furthermore, in elderly patients with trauma, particularly in those with severe trauma, factors such as age and underlying conditions contribute to a higher rate of inability to undergo surgery or intervention compared to non-elderly patients [25]. This may explain why elderly patients often do not undergo surgical treatment or invasive interventions despite having sustained severe injuries.

In this analysis, elderly patients showed significantly lower survival rates in cases of severe trauma compared to their non-elderly counterparts. We also observed a notable reduction in the frequency of surgical or embolization interventions performed within the group with severe trauma, which persisted even in the elderly patient’s cohort. The increased intervention rates in moderate cases but decreased rates in severe cases among the elderly may be attributed to the frail physiological states of these patients. Factors such as pre-existing conditions, which can deteriorate rapidly, thereby elevating the risks associated with invasive treatments, play a significant role in this phenomenon. Another potential cause is the higher rate of under-triage among elderly patients with severe traumatic injuries in the prehospital setting. Consequently, these patients may be transferred to hospitals lacking advanced surgical or embolization interventions for trauma. Hoyhe et al. reported that elderly major trauma patients are at greater risk of under-triage [26]. This under-triage of elderly patients could result in them being less likely to be triaged to major trauma centers. Therefore, managing severe trauma in elderly patients may necessitate prompt transfers to advanced medical facilities and treatment approaches that accommodate their unique physiological profiles.

During the EMS phase, ISSs can be helpful for rapidly evaluating injury severity, thus enabling prioritized care for those with the most severe injuries [27,28]. The ISS also aids in determining the need for patient transfer to suitable medical facilities, with higher scores often prompting rapid relocation to general hospitals or specialized trauma centers [28,29]. However, this study demonstrates that elderly trauma patients have higher rates of surgery or embolization compared to their non-elderly counterparts, except in instances of severe trauma. Furthermore, within equivalent severity levels, elderly patients exhibit lower survival rates. These findings highlight the need for a specialized approach to trauma management in elderly patients, distinct from the strategies employed for younger patients.

This study had several limitations. First, the data were collected over a single year, which may have reduced our ability to capture longitudinal trends. Second, the exclusion criteria may introduce certain biases. For example, patients with incomplete medical records or those transferred from other facilities were excluded, potentially resulting in selection bias. These biases are important to consider when interpreting the results. Third, the retrospective observational nature of the study makes it susceptible to potential biases, including selection bias and reporting bias. Although we utilized nationwide data, there is still a risk of bias. Therefore, the results should be interpreted with caution. Fourth, our study utilized data from the South Korean EMS system, and the characteristics of the patient population may differ from those in other regions. Consequently, the generalizability of our findings to other populations or healthcare systems may be constrained. To enhance the robustness of our conclusions, future research should be conducted in diverse settings to further validate our findings. Finally, while the ISS primarily focuses on assessing physical injuries, other factors such as preexisting chronic conditions, overall health status, and cognitive decline can also significantly impact treatment outcomes in the elderly patient demographic.

## 5. Conclusions

The ISS may correlate with survival outcomes in elderly trauma patients. However, decisions on therapeutic options based exclusively on ISS are of limited value in severe trauma cases. Additional assessment tools, such as the RTS, should be considered for a more comprehensive therapeutic. To enhance the severity assessment of elderly trauma patients, the development of trauma severity tools based on both anatomical and physiological abnormalities is required.

## Figures and Tables

**Table 1:** Demographic characteristics of the study population, divided by age.

Variables	Total Patients	Elderly Patients with Trauma	Non-Elderly Patients with Trauma	*p* Value
	(n = 16,336)	(n = 4886)	(n = 11,450)	
Sex, male %	11,047 (67.6%)	2793 (57.2%)	8254 (72.1%)	<0.001
Age years	55.0 [39.0–68.0].	75.0 [69.0–81.0].	47.0 [32.0–56.0]	<0.001
Metropolitan	7221 (44.2%)	2358 (48.3%)	4863 (42.5%)	<0.001
Call to ER arrival, minutes	31.0 [23.0–43.0].	31.0 [24.0–44.0].	30.0 [23.0–43.0]	<0.001
Hospital level				0.026
Level I	2223 (13.6%)	658 (13.5%)	1565 (13.7%)	
Level II	7257 (44.4%)	2247 (46.0%)	5010 (43.8%)	
Level III or trauma center	6856 (42.0%)	1981 (40.5%)	4875 (42.6%)	
Mechanism of injury				
Traffic accident	6906 (42.3%)	1792 (36.7%)	5114 (44.7%)	<0.001
Fall or slide	7132 (43.7%)	2751 (56.3%)	4381 (38.3%)	
Contusion	1147 (7.0%)	178 (3.6%)	969 (8.5%)	
Penetrating or mechanical injury	1151 (7.0%)	165 (3.4%)	986 (8.6%)	
Transfusion	1736 (10.6%)	614 (12.6%)	1122 (9.8%)	<0.001
ISS				<0.001
–Mild (1–8)	11,141 (68.2%)	2969 (60.8%)	11,141 (68.2%)	
–Moderate (9–15)	2903 (17.8%)	1105 (22.6%)	2903 (17.8%)	
–Severe (≥16)	2292 (14.0%)	812 (16.6%)	2292 (14.0%)	
Outcomes				
Rate of Surgery or embolization	3576 (21.9%)	1071 (21.9%)	2505 (21.9%)	0.969
Survival to discharge	14,826 (90.8%)	4117 (84.3%)	10,709 (93.5%)	<0.001

ER, emergency room; ISS, injury severity score.

**Table 2:** Multivariable logistic regression analysis by injury severity score for elderly patients with trauma.

Outcome	Groups	Reference = Mild (ISS 1–8)				Reference = Moderate (ISS 9–15)			
		OR *	95% CI		*p*	OR *	95% CI		*p*
Rate of Surgery or embolization	ISS 1–8	1.00				0.121	0.100	0.146	<0.001
	ISS 9–15	8.298	6.856	10.043	<0.001	1.00			
	ISS ≥16	7.027	5.597	8.822	<0.001	0.847	0.690	1.040	0.113
Survival to discharge	ISS 1–8	1.00				6.975	5.032	9.669	<0.001
	ISS 9–15	0.143	0.103	0.199	<0.001	1.00			
	ISS ≥16	0.019	0.013	0.026	<0.001	0.130	0.102	0.165	<0.001

* Adjusted odds ratios for age, sex, metropolitan area, time from call to ER arrival, hospital level, mechanism of injury, injury severity score, and need for blood transfusion. ISS, injury severity score; OR, adjusted odds ratio; CI, confidence interval.

**Table 3:** Multivariable logistic regression analysis by injury severity score for non-elderly patients with trauma.

Outcome	Groups	Reference = Mild (ISS 1–8)				Reference = Moderate (ISS 9–15)			
		OR *	95% CI		*p*	OR *	95% CI		*p*
Rate of Surgery or embolization	ISS 1–8	1.00				0.136	0.119	0.155	<0.001
	ISS 9–15	7.373	6.443	8.436	<0.001	1.00			
	ISS ≥16	13.242	11.291	15.532	<0.001	1.796	1.530	2.109	<0.001
Survival to discharge	ISS 1–8	1.00				12.263	8.324	18.066	<0.001
	ISS 9–15	0.082	0.055	0.120	<0.001	1.00			
	ISS ≥16	0.015	0.010	0.022	<0.001	0.183	0.146	0.229	<0.001

* Adjusted odds ratios for age, sex, metropolitan area, time from call to ER arrival, hospital level, mechanism of injury, injury severity score, and need for blood transfusion. ISS, injury severity score; OR, adjusted odds ratio; CI, confidence interval.

**Table 4:** Multivariable logistic regression for outcomes of patients with trauma according to injury severity score.

Outcomes	Groups	Mild (ISS 1–8)				Moderate (ISS 9–15)				Severe (ISS ≥ 16)			
		OR *	95% CI		*p*	OR *	95% CI		*p*	OR *	95% CI		*p*
Rate of Surgery or embolization	Non-elderly	1.00				1.00				1.00			
	Elderly	1.201	0.945	1.526	0.135	1.351	1.035	1.763	0.027	0.623	0.465	0.834	0.002
Survival to discharge	Non-elderly	1.00				1.00				1.00			
	Elderly	0.689	0.326	1.457	0.330	0.856	0.542	1.350	0.503	0.705	0.522	0.953	0.023

*Adjusted odds ratios for sex, age, metropolitan area, time from call to ER arrival, hospital level, mechanism of injury, and need for blood transfusion. ISS, injury severity score; OR, adjusted odds ratio; CI, confidence interval.

## Data Availability

The authors used data from a database that has been made available by the Korea Disease Control and Prevention Agency, which holds authority over the Severe Trauma Registry dataset in Korea. Access to this dataset requires permission from the organization, which can be requested at: http://www.kdca.go.kr/injury/(accessed on 9 September 2023).

## Supplementary Materials

The following supporting information can be downloaded at: https://www.mdpi.com/article/10.3390/jpm14070674/s1, Table S1: Criteria for detailed classification of causes of trauma; Table S2: Definitions of Levels of Hospital Care.
