# Supplementary material for: Prognostic Association between Injury Severity Score and the Outcomes of Elderly Patients with Trauma in South Korea
Source: J Pers Med. 2024 Jun 23;14(7):674. doi: 10.3390/jpm14070674 (PMC11277643; doi:10.3390/jpm14070674)
Supplement: Supplementary file 1 [file jpm-14-00674-s001.zip › jpm-3040844-supplementary.pdf]

Table S1. Criteria for detailed classification of causes of trauma.

**Category**	**Detailed Description**
1) Transport Accidents	- Traffic accidents, damages related to transport vehicles, including intentional incidents.<br>- Collisions involving motorized vehicles in all places.<br>- Collisions involving pedestrians and non-motorized transport vehicles or bicycles on roads and off-road traffic areas.<br>- Excludes accidents involving transport vehicles where the causes are unrelated to automobiles and their movements (People in wheelchairs are considered pedestrians).
2) Falls and Slips	- Tripping or slipping on the same level, falling from buildings, slipping on stairs, falling downstairs.
3) Blunt Trauma	- Damage caused by collisions or impacts.<br>- Being hit by a bat, hitting the head against a wall, contact with thrown objects, bodily crush (trapped, run over, etc.), crushing.<br>- Includes hitting a traffic light while riding inline skates.<br>- Excludes continuous friction, bites by animals, and scratches.
4) Penetrating Wounds	- Damage caused by stabbing or piercing forces.<br>- Scratches, cuts, tears, penetrations, wounds, stabbings, hit by firearms (using gunpowder), hit by air guns, hit by arrows from bows, bullets from spring weapons.<br>- Bites by humans, animals, dogs, and snakes, stung by bees.
5) Machinery	- Impact from explosions, contact with machinery, crushing by machinery.

Table S2. Definitions of Levels of Hospital Care

**Level of Hospital Care**	**Terminology and Definitions**
First-level Hospital	- Few specialties—mainly internal medicine, obstetrics and gynecology, pediatrics, and general surgery.<br>- Often only one general practice physician or a nonphysician clinician.<br>- Limited laboratory services are available for general analysis but not for specialized pathological analysis.<br>- 50–250 beds.
Second-level Hospital	- More differentiated by function, with as many as 5 to 10 clinical specialties.<br>- 200–800 beds.
Third-level Hospital	- Highly specialized staff and technical equipment—for example, cardiology, intensive care unit, and specialized imaging units.<br>- Clinical services are highly differentiated by function.<br>- Teaching activities in some facilities.<br>- 300–1,500 beds.
Trauma center	the Korean government initiated a new Korean trauma system by establishing 17 regional trauma centers, each equivalent to a level-1 trauma center in the Unite states of America
